# Supplementary material for: Start learning coding without computers? A case study on children’s unplugged gamified coding education tool with explanatory sequential mixed method
Source: PLoS One. 2025 Sep 3;20(9):e0330896. doi: 10.1371/journal.pone.0330896 (PMC12407412; doi:10.1371/journal.pone.0330896)
Supplement: S1 File — Appendices A and B. (DOCX) [file pone.0330896.s001.docx]

Appendix A. (Summary of Construct Items and Sources).

| **Construct** | **Code** | **Questionnaire Item** | **Source** |
| --- | --- | --- | --- |
| Role-Playing (RP) | RP1 | Role-playing allows me to understand the story of the game. | [68] |
|  | RP2 | I feel more interested after understanding the story of the game. |  |
|  | RP3 | Role-playing teaches me a lot about game play. |  |
|  | RP4 | Playing as a hero makes the game funnier. |  |
| Reward (RE) | RE1 | Rewards are easy to get in the game. |  |
|  | RE2 | I feel more motivated after getting rewards. |  |
|  | RE3 | Getting a reward is an important reason to motivate me to play the game. |  |
|  | RE4 | Getting rewards makes the game funnier. |  |
| Challenge (CH) | CH1 | The gameplay is very challenging. |  |
|  | CH2 | I feel more motivated after be challenged in the game. |  |
|  | CH3 | Challenges make me familiar with the game operation. |  |
|  | CH4 | Challenges make the game funnier. |  |
| Cooperation (CO) | CO1 | I am happy to cooperate with my friends in the game. |  |
|  | CO2 | Cooperation makes it easier for me to win the game. |  |
|  | CO3 | Cooperation helps me to develop my gaming skill. |  |
|  | CO4 | Cooperation makes the game funnier. |  |
| Flow (FL) | FL1 | I don’t notice time passing. | [42] |
|  | FL2 | I have no difficulty concentrating. |  |
|  | FL3 | I am totally absorbed in what I am doing. |  |
|  | FL4 | I feel just the right amount of challenge. |  |
|  | FL5 | I know what I have to do each step of the way. |  |
| Learning Engagement (LE) | LE1 | After playing the game, I am willing to take the initiative to analyze problems. | [69] |
|  | LE2 | After playing the game, I am willing to take effective learning. |  |
|  | LE3 | After playing the game, I am willing to solve practical learning problems. |  |
|  | LE4 | After playing the game, I am willing to engage in knowledge acquisition. |  |

Appendix B. (Summary of Interview Questions in Qualitative Study).

| **Topic / Theme** | **Quantitative Finding / Hypothesis Testing** | **Interview Question** |
| --- | --- | --- |
| Role-Playing | Role-playing -> Flow (Accepted)  Role-playing -> Learning engagement (Rejected) | 1. How does it feel to play a hero in the game?  2. Does playing as a hero help you understand what you are learning in the game? Why? |
| Reward | Reward -> Flow (Rejected)  Reward -> Learning engagement (Accepted) | 1. How does it feel to get rewards while playing games?  2. How does getting rewards help you study harder in the game? |
| Challenge | Challenge -> Flow (Rejected)  Challenge -> Learning engagement (Rejected) | 1. Did this game provide you with a challenge? How did you feel about it?  2. Do you think challenges motivate you to learn more in games? Why? |
| Cooperation | Cooperation -> Flow (Accepted)  Cooperation -> Learning engagement (Accepted) | 1. How does cooperation in games help you solve problems?  2. How does cooperation improve your learning efficiency in the game? |
