# Supplementary material for: Start learning coding without computers? A case study on children’s unplugged gamified coding education tool with explanatory sequential mixed method
Source: PLoS One. 2025 Sep 3;20(9):e0330896. doi: 10.1371/journal.pone.0330896 (PMC12407412; doi:10.1371/journal.pone.0330896)
Supplement: Attachment — Submitted filename: Response to Reviewers.docx [file pone.0330896.s004.docx]

| Report: | | Response to the reviewers’ comments and suggestions | |  | |  | |
| --- | --- | --- | --- | --- | --- | --- | --- |
| Date: | | 17/FEB/2025 | |  | |  | |
| **Rev** | **Comments and suggestions** | | **Section/page** | | **Responses/ Action taken by Author(s)** | |  |
| Editor | 1 | I recommend organizing your discussion around specific themes. | Section 6 / P-20 | | The discussion has been reorganized according to seven themes, corresponding to the above integration Table 10. | |  |
|  | 2 | The "design content" column in Table 1 needs to be more technical. The phrase "children will be rewarded for trying the games" is merely a noun definition of "reward". In the design section, you should explicitly outline the design logic and implementation criteria of the reward system, such as which types of rewards will be given for specific user behaviors and what those rewards are. Please revise accordingly. | Section 4.2 / P-10 | | Revised the columns of Table 1 and explained the reward design logic in detail. | |  |
|  | 3 | In your hypotheses, you’ve prefixed each game element with the word “gamified”, such as “gamified challenges”, “gamified cooperation”, and “gamified role-playing”. I believe the term "gamified" should be removed. | Section 3 / P-8 | | The word “gamified” was removed from all contents related to the hypotheses in the context. | |  |
|  | 4 | The description of Table 5 is flawed. There are mistakes. Please revise. | Section 5.1.1 / P-13 | | Revised. | |  |
|  | 5 | You mention that “all variables have sufficient discriminant validity". Please provide the benchmark for clarity. | Section 5.1.1 / P-13 | | All benchmarks related to discriminant validity have been re-stated. | |  |
|  | 6 | Please expand on the descriptions of Table 5 and Table 6. | Section 5.1.1 / P-13 | | Expanded. | |  |
|  | 7 | The headers in the first and last columns of Table 7, “hypothesis/relationship” and “decision,” are inaccurate. These should be revised. | Section 5.1.2 / P-14-15 | | Headers in Table 7-9 are revised. | |  |
|  | 8 | The header in Table 1 needs revision. The content in the first column should be summarized as something like “game elements”, not "gamification." Additionally, the wording of “design content” may not be appropriate. Please revise. | Section 4.2 / P-10 | | Revised. | |  |
|  | 9 | Table 2 needs headers. You need to include column headers so that we know how to interpret the data. For example, with a header of "frequency", we understand that 63 and 91 should be entered; with a header of "percentage", we know to enter 21.36% and 30.85%. What should the headers for “age” and “gender” be? Please add. | Section 4.3.1 / P-11 | | Added. | |  |
|  | 10 | In Table 2, is it “coding experience” or “coding education experience”? The latter refers to the experience of teaching coding, right? Please revise. | Section 4.3.1 / P-11 | | Revised. | |  |
|  | 11 | Please avoid using symbols like “&” in the main text. The text should only contain English letters and Arabic numerals. For instance, it should be “Fornell and Larcker criterion”, not “Fornell & Larcker criterion.” | Section 5.1.1 / P-13 | | “&” has been uniformly replaced by “and” in the context. | |  |
|  | 12 | Please ensure consistency in the use of the term "Fornell-Larcker criterion". Currently, it appears interchangeably as "Fornell-Larcker criterion" and "Fornell and Larcker criterion". | Section 5.1.1 / P-13 | | It has been determined to be the “Fornell-Larcker criterion.” | |  |
|  | 13 | When using abbreviations for the first time, provide their full form, e.g., S-O-R theory. | Abstract / P-1 | | Revised in context. | |  |
|  | 14 | Please verify the use of single and double quotation marks to ensure they are applied correctly. | Full text | | Double quotes have been used throughout the text. | |  |
|  | 15 | Please check whether capitalization is needed for any of the words in the table. Currently, you have expressions like “One Year and Above”. | Full text | | The capitalization of text in all tables has been unified. | |  |
|  | 16 | Ensure consistency in the word forms for the elements in Table 10. For example, “to be hero” is a verb, while “teamwork” is a noun. | Section 5.3 / P-16 | | The categories in Table 10 have been unified into noun forms. | |  |
|  | 17 | It’s recommended to adjust the font in Figure 1 to match the font used in the main text. Optional. | Section 3 / P-6; Section 4.1 / P-9 | | The font in Figures 1 and 2 has been unified with the font of the main text. | |  |
| Reviewer #1 | 1 | Expand the Qualitative Analysis:  Provide more detailed insights from the interviews, including direct quotes or examples of children’s responses. This would make the qualitative findings more vivid and compelling. | Section 6 / P-20 | | Direct quotations from participants reinforced the credibility of findings and the effectiveness of explanations across all discussion themes. | |  |
|  | 2 | Enhance the Discussion of Limitations:  Discuss the potential impact of cultural context, sampling limitations, and implementation challenges on the study’s findings. This would provide a more nuanced understanding of the study’s scope and generalizability. | Section 7.3 / P-22 | | The limitations section was strengthened by adding explanations of cultural differences and sampling diversity limitations. | |  |
|  | 3 | Clarify Practical Implications:  Provide specific recommendations for educators and developers on how to implement the findings in real-world settings. For example, what are the key design principles for creating effective unplugged gamified coding tools? | Section 7.2 / P-21 | | The specific design experience and effective gamification elements gained from this study are supplemented to enhance practical significance. | |  |
|  | 4 | Discuss Effect Sizes in Context:  Explain the practical significance of the effect sizes observed in the study. For instance, how might a “large” effect size translate into measurable improvements in children’s learning outcomes? | Section 6 / P-19 | | The effect size description and explanation has been incorporated into all themes in the discussion. | |  |
|  | 5 | Explore Interactions Between Gamification Elements:  Investigate how different gamification elements (e.g., rewards, role-playing, cooperation) interact with each other to influence flow experience and learning engagement. | Section 6 / P-20 | | A paragraph was added to the discussion detailing the interrelationships of gamification elements and their impact on learning engagement. | |  |
| Reviewer #2 | 1 | This paper explores gamified unplugged coding education for young learners, grounded in Stimulus-Organism-Response (S-O-R) theory. While the research topic is timely and relevant, the manuscript has several major weaknesses that must be addressed before it can be considered for publication. The theoretical contribution, though well-articulated, lacks sufficient critical engagement with prior work on gamification and coding education, especially in non-digital learning environments. Additionally, the research problem and hypotheses are formulated in a way that assumes gamification inherently improves learning outcomes rather than critically interrogating the conditions under which different elements succeed or fail. This assumption weakens the theoretical framing and should be revisited. | Section 1 / P-3 | | Two paragraphs were added to the introduction to critically discuss the current status and problems of unplugged coding education and related research, in order to emphasize the necessity of this study. | |  |
|  | 2 | Methodologically, the study has some problems, from sampling limitations and potential biases that undermine the validity of its findings. The sample consists of only two schools in Nanjing, raising concerns about generalizability. There is no discussion of socio-economic or educational background factors, which are crucial in evaluating the effectiveness of gamified learning. Moreover, the PLS-SEM analysis lacks depth, particularly in its treatment of mediation effects and model robustness. The lack of a pre-test/post-test design makes it impossible to determine whether the observed effects are due to actual learning improvements or merely engagement during the gamified activity. The qualitative component is also underdeveloped—thematic analysis is presented descriptively rather than critically. The study would benefit from a more rigorous qualitative coding approach supported by inter-rater reliability checks. | Section 7.3 / P-22 | | The regret that a more comprehensive research design was not fully implemented in the methodology has been mentioned in Limitations and future directions, and it is hoped that they will be compensated in future research. | |  |
|  | 3 | A major issue is the superficial treatment of negative or neutral outcomes. The study claims that role-playing and cooperation significantly enhance the flow of experience and learning engagement, but it does not account for the weaker impact of challenges and rewards. Instead of reporting this finding, the authors should analyze why these elements underperformed—perhaps due to poor design, cognitive overload, or lack of individual motivation factors. Without a clear discussion of these inconsistencies, the conclusions feel overstated. Additionally, the long-term impact of the intervention is completely ignored. Gamification often produces short-term engagement spikes that do not translate into sustained learning benefits, a limitation that should have been explicitly addressed. | Section 7.2 / P-21 | | The specific design experience and effective gamification elements gained from this study are supplemented to enhance practical significance. | |  |
|  | 4 | The practical implications of the study are also overstated. The "Coding Adventure" prototype is not rigorously tested against existing educational tools, making it difficult to determine whether its benefits are due to the gamification elements themselves or simply the novelty of the experience. There is no comparative analysis with digital coding tools or traditional instructional methods, which is a major oversight. Furthermore, the study ignores a crucial stakeholder group: parents and teachers. Given the emphasis on unplugged learning as an alternative to screen-based education, the study should include perspectives from educators and parents to evaluate whether such an approach is feasible for widespread adoption. | Section 7.3 / P-22 | | The researchers included a comparison between the unplugged coding teaching tool and traditional teaching tools in the introduction, and criticized the shortcomings of the prototype design in the qualitative results and discussion sections. At the end, they also pointed out the lack of teacher and parent perspectives in the limitations. | |  |
|  | 5 | In conclusion, this paper requires major revisions before it can be considered for publication. The theoretical framing should be refined to critically engage with prior research, rather than assuming gamification’s benefits. The methodology must address its generalizability issues, incorporate a more rigorous qualitative approach, and critically assess underperforming gamification elements. Finally, the practical contributions need substantial revision, with comparative analysis and long-term evaluation. Until these concerns are addressed, the study remains an interesting but incomplete contribution to gamified coding education. | Full text / Section 6 / P-20 | | The researchers integrated all previous revision suggestions, focused on critically discussing the successful experiences and shortcomings of prototype development, revised the research methodology from a more comprehensive perspective, and strengthened the summary of significances and limitations. | |  |
