# Supplementary material for: Start learning coding without computers? A case study on children’s unplugged gamified coding education tool with explanatory sequential mixed method
Source: PLoS One. 2025 Sep 3;20(9):e0330896. doi: 10.1371/journal.pone.0330896 (PMC12407412; doi:10.1371/journal.pone.0330896)
Supplement: Attachment — Submitted filename: Manuscript - revisions.docx [file pone.0330896.s003.docx]

**Start Learning Coding without Computers? A Case Study on Children’s Gamified Coding Education Tool with Explanatory Sequential Mixed Method**

**Miao Huang^1^ , Lei Wang ^2🞸^**

1. School of Animation and Digital Arts, Communication University of China, Nanjing, China. E-mail: huangmia@cucn.edu.cn, <https://orcid.org/0000-0003-1762-2239>

2. School of Animation and Digital Arts, Communication University of China, Nanjing, China. E-mail: wanglei@cucn.edu.cn, https://orcid.org/0000-0002-0663-5870

*Correspondence Author

Abstract

Gamification is believed to improve the teaching effectiveness of children’s coding education tools, but Chinese parents have stricter demands for protecting children’s health. The researchers added role-playing, rewards, challenges, and collaboration as gamification elements to the design of unplugged children’s coding education tools to test their effects on children’s flow experience and learning engagement. The researchers of this study designed an unplugged coding education tool called “Coding Adventure” based on the research objectives, and used the explanatory sequential mixed method to conduct a quantitative survey on 295 Chinese primary school students aged 8-10 years old, and conduct qualitative interviews with 12 of them. The results of this study show that role-playing and cooperation effectively enhance children’s flow experience when engaging in coding education tools. Children role-play heroes and engage in “real fights,” engage in teamwork and communicate with others, which can bring them into a flow experience. The flow experience effectively enhances children’s learning engagement. The main reasons are the immersion, upgrade experience, practicality, goal-orientedness, teamwork and partner’s suggestions brought by gamification elements. Moreover, rewards and cooperation also directly positively influence children’s learning engagement. Intrinsic and extrinsic rewards, engagement in teamwork and receiving encouragement and suggestions from interactions with classmates are thought to increase children’s motivation to learn. In addition, the flow experience is found to have mediating effects in the process of role-playing and cooperation influences children’s learning engagement. This study examined the effects of diverse gamification elements on children’s flow experience and learning engagement in an unplugged gamified coding education tool under the framework of the stimulus-organism-response theory. This is of great significance to future coding education tool developers, which has reference significance.

Keywords:

Coding education; gamification; children; flow experience; learning engagement

1. Introduction

Computational thinking (CT) has become a new standard for global users to integrate into digital products and information services (Vyas, 2022). In China, where the public pays more attention to children’s education, educators are gradually attaching importance to adding coding education to kindergartens and primary schools to popularise the cultivation of CT (Folgieri et al., 2019). Such coding education is often integrated with gamification to enhance the teaching effect and stimulate children’s CT to adapt to the future environment of robots and artificial intelligence (Folgieri et al., 2019).

Gamified learning refers to educators adding game-specific design elements to non-game educational situations (Sanchez et al., 2020). The introduction of gamification mechanisms can not only stimulate students’ learning engagement but also strengthen their motivation to solve problems (Krath et al., 2021). However, Chinese parents generally do not support computer-based teaching methods for children, believing that these methods will affect children’s visual health and challenge children’s self-control (Dong et al., 2020). In addition, children may also experience loneliness due to long hours of computer and online learning, leading to their mental health problems (McMahon et al., 2021). Therefore, a coding system of unplugged flowblocks for children was proposed to enhance children’s self-efficacy (Threekunprapa & Yasri, 2020).

In essence, unplugged physical coding education is a compromise development for children, but it has been widely praised by educators for popularising coding knowledge among younger age groups (Babić & Čičin-Šain, 2023).In today’s educational contexts, visual coding education tools represented by Scratch and ScratchJr have limited effectiveness for children, especially when teachers do not predominate complete coding skills (Chou, 2020).Therefore, unplugged coding courses can help ordinary teachers with non-digital majors expand their digital literacy in children’s basic education.

In ordinary educational contexts, teachers can consider arranging unplugged game scenes and guiding children to experience the introductory knowledge of CT (Akiba, 2022).In physical educational contexts, education tool designers can further take advantage of children’s preferences for cartoon images, hero stories, props and small animals, and integrate these elements into education games (Lee & Junoh, 2019).Such education tools can be further developed into treasure hunting games, allowing children to stimulate their inherent interest in exploration and use the classroom environment to recreate game scenes (Lee et al., 2022).

Although previous researchers have proposed various solutions for children’s unplugged gamified coding education tools, there are few cases of integrating them into tests. Research on the effects of such tools on children is even scarcer. Therefore, this study plans to test the effects of gamification on children’s learning engagement by using a prototype that conforms to the characteristics of unplugged and gamified environments so as to understand how different gamification elements work in an unplugged real environment.

2. Literature review
   2.1. Gamification elements (role-playing, reward, challenge, cooperation)

Gamification encompasses many design elements, many of which often work together (An, 2020). In the case of gamification applied to education, role-playing, reward, challenge, and cooperation have received the focus of researchers as gamification design elements (Hernández et al., 2021; Fantazir & Bartley, 2021; An, 2020). Combining gamified role-playing with reward, challenge, and cooperation for education management can effectively stimulate primary school students’ enthusiasm for learning (Chen et al., 2022).

Role-playing can add fun, positive and social characteristics to education (Aura et al., 2023). In the context of role-playing, instructional designers can incorporate diverse reward elements such as points, medals, and rankings, and can also add challenges and punishments as motivational elements (Zainuddin & Keumala, 2021). Rewards and punishments (feedback), as important components of the gamified incentive system, effectively promote children’s behavioural motivation (Lomos et al., 2023). Challenge-based and achievement-based gamified design focuses on encouraging users to overcome difficulties, make progress, and ultimately feel empowered (Legaki et al., 2020). The advantages of gamified cooperation in the education field encourage students to engage in a cooperative environment through role-playing mutual assistance, resource sharing, teamwork and even moderate competition (Riar et al., 2022).

2.2. Flow experience in gamified education

According to the flow theory proposed by Csikzentimihalyi (1975), flow refers to “the holistic sensation that people feel when they act with total involvement.” (pp. 36). Flow experience can be further understood as the best psychological state felt by an individual, an optimal experience that maintains an individual’s state of excitement or enjoyment (Csikszentmihalyi, 1990). Flow experience is a psychological activity highly related to learning experience (Oliveira et al., 2021). Gamification is considered one of the most effective ways to stimulate flow experiences (Bitrián et al., 2020). Gamified education improves students’ flow experience in classroom learning, thereby improving their learning results (Zhao & Li, 2020). Educators or designers cannot provide students with flow, but they can provide students with gamification elements to help them enter the flow state (Luo, 2022).

2.3. Children’s learning engagement

Learning engagement, as an active and fulfilling learning state, is widely recognised as a key factor in students’ academic success (Luo et al., 2021; Wang et al., 2021). Children’s enthusiasm for learning in primary school has been found to predict their future academic achievement, work status and life happiness (Carmona-Halty et al., 2021). In China, even primary school students’ learning engagement results are regarded as an important way to improve their social status, closely affecting their future development (Zhang et al., 2020; Huang et al., 2020). Therefore, the learning engagement of Chinese primary school students is regarded as an extremely important observational indicator. In the platform construction of computer science education, educators use gamification technology to develop learning environments for students to attract students and make them actively engage in interesting, exciting, and engaging courses (Papadakis & Kalogiannakis, 2019). Moreover, for younger children, teachers’ interaction and guidance in the classroom environment are closely related to children’s engagement in education activities (Pianta et al., 2020).

3. Theoretical foundation and hypotheses development

The stimulus-organism-response (S-O-R) theory was founded in 1974 (Mehrabian & Russell, 1974). The original intention of creating the theory was to study the consumption behaviour of individuals (Jacoby, 2002, Vieira, 2013). Since then, the S-O-R theory has also been extended to the study of flow experience stimulated by gamification, proving that flow experience mediates the relationship between gamification and learning engagement (Thomas & Baral, 2023). On this foundation, the researchers applied the four gamification elements (role-playing, reward, challenge and cooperation) suitable for children’s unplugged coding education tool design in the literature review to refine the S-O-R theory based on gamification flow research, and obtained the following theoretical framework. Figure 1 shows theoretical framework of this study.

**Figure 1.** Theoretical Framework

Role-playing can provide users with imaginative immersion, allowing them to be fully immersed in the experience through role identification and imaginary cognition (Shroff et al., 2019). Role-playing, game plot and narrative constitute an immersive gaming experience that draws users into the game world and evokes a state of complete immersion in them (Wallius et al., 2023). Therefore, the following hypothesis is proposed:

**H1: Role-playing positively influences children’s flow experience in the coding education tool.**

Flow theory predicts that the balance between gamified task difficulty and individual operating skill will lead to high levels of intrinsic reward, ultimately motivating the allocation of cognitive control (Huskey et al., 2018). Such intrinsic rewards encourage individuals to focus entirely on the activity itself and gain a strong sense of inner happiness, thereby achieving the flow experience (Mao et al., 2020). Therefore, the following hypothesis is proposed:

**H2: Rewards positively influence children’s flow experience in the coding education tool.**

Gamified challenges should match the user’s ability level, provide clear goals and immediate feedback to promote the flow state within the game (Ofosu-Ampong, 2020). The study by Anunpattana et al. (2021) used flow theory to explain the process of gamified challenges affecting students’ abilities, in which they detected that as the challenge increases and abilities increase, students’ flow experience will further enter the arousal zone. Therefore, the following hypothesis is proposed:

**H3: Challenges positively influence children’s flow experience in the coding education tool.**

In multiplayer games, effective communication and teamwork, and sharing relevant information about game tasks with team members, effectively stimulate group flow in cooperative games (Kaye, 2016). Compared with the competitive virtual environment experience, the cooperative mode stimulates higher levels of emotion and decision-making challenges, and also promotes users’ flow experience (Bian et al., 2023). Therefore, the following hypothesis is proposed:

**H4: Cooperation positively influences children’s flow experience in the coding education tool.**

Students experienced a flow state while applying learning games with virtual reality technology, which provided them with a high sense of immersion and presence, promoting their learning engagement (Bodzin et al., 2020). Park (2020) found that learning flow is a predictor of learning engagement and teaching presence in coding education. Therefore, the following hypothesis is proposed:

**H5: The flow experience positively influences children’s learning engagement in the coding education tool.**

The quasi-experimental study by Yang et al. (2023) obtained comparative results, proving that role-playing-based CT education strategies show a significant effect in promoting students’ learning engagement and motivation. In addition, role-playing strategies have also been shown to enhance students’ learning engagement and critical thinking in online teaching (Berry & Kowal, 2022). Therefore, the following hypothesis is proposed:

**H6: Role-playing positively influences children’s learning engagement in the coding education tool.**

Students gain inner pleasure and satisfaction through learning activities and feel intrinsic rewards; they also feel extrinsic rewards by receiving praise and encouragement from society and family, as well as scholarships or career development given by others (Huang & Yang, 2021). When children complete their learning progress, the gamification system quickly gives back rewards to provide a sense of achievement, thereby stimulating children’s stronger desire to learn (Qian et al., 2023). Therefore, the following hypothesis is proposed:

**H7: Rewards positively influence children’s learning engagement in the coding education tool.**

Compared with traditional education methods, challenge-based gamified education has a more positive effect on students’ learning (Legaki et al., 2020). The use of challenge-based gamified learning methods by students effectively improved their academic performance levels and overall motivation (Kaya & Ercag, 2023). Therefore, the following hypothesis is proposed:

**H8: Challenges positively influence children’s learning engagement in the coding education tool.**

When applying smart classrooms as teaching environments, cooperative learning strategies have been proven to improve primary school students’ overall learning engagement (including behavioural engagement and emotional engagement) (Qin et al., 2020). In neuroscience, researchers have also found that social closeness (e.g., liking for each other) between learners is reflected in intra-group neural synchrony and co-varies with learning engagement, which suggests that successful collaboration promotes the establishment of engagement consensus (Pan et al., 2023). Therefore, the following hypothesis is proposed:

**H9: Cooperation positively influences children’s learning engagement in the coding education tool.**

4. Methodology
   4.1. Research design

Considering that the subjects of this study are still young with incomplete understanding and expression abilities, the results obtained by a single research method may not be representative. Therefore, this study adopted the explanatory sequential mixed method to minimise the possible errors caused by a single method and combined quantitative and qualitative methods to capture the reasons behind the phenomenon (Wipulanusat et al., 2020). The explanatory sequential mixed method is divided into two stages: a quantitative survey and qualitative interviews in this study (Sovacool et al., 2018). The study has been assigned study protocol code 2023/CUCN/IRB/0009, which should be used for all communications related to this study. Recruitment for this study will begin on 3rd March 2023 and end on 2nd March 2024. Participants provided verbal informed consent, received verbal approval from the school and guardian teachers, and consent was obtained from parents or guardians. In the first stage, quantitative data is obtained to determine preliminary results and explained in detail through the quantitative results (Creswell, 2014). Due to the need to observe children’s feedback on specific coding education tools, the researchers designed a prototype for coding education that meets the research objectives in the preparation phase. Figure 2 shows the detailed steps of this study.

**Figure 2.** The Research Process Based on the Explanatory Sequential Mixed Method (Creswell, 2014)

4.2. Prototype design and application

Based on the research objectives mentioned above, the researchers have sorted out the currently common gamified coding education tools. Most of them cannot simultaneously achieve the experimental requirements that this study hopes to achieve, and some tools involve copyright issues. Therefore, the researchers designed a prototype that fully meets the experimental requirements, called “Coding Adventure”, which achieves the established gamification functions through the following design content (Table 1).

**Table 1.** Design of “Coding Adventure” Prototype

| **Game Element** | **Specific Game Rules** |
| --- | --- |
| Role-playing | Children play the role of knights; they can wear safe props as equipment; they will be told the adventurous game story before the game starts; there are exploration and rescue as mission goals. |
| Reward | Children will receive small dolls as rewards after passing levels; once children defeat monsters, the level and skills of their characters will be upgraded; completing ordinary tasks will get one doll, and completing difficult tasks will get double the number of dolls. |
| Challenge | Each level has multiple routes; there are obstacles and monsters as obstacles to pass the level; the difficulty will increase as the game progresses; the monsters have special counterattacks. |
| Cooperation | Allows multiple players at the same time; children can plan with their teammates before the game starts; children can ask for help from classmates outside the game when they encounter difficulties; classmates can watch and encourage; teachers will provide information tips. |

After the prototype design of “Coding Adventure” was completed, the researchers applied to enter twelve classes in two primary schools for testing in Nanjing, China. The entire testing process was approved by the primary schools and conducted openly under the supervision of teachers in the schools. The researchers used positive guidance and kind language throughout the test to ensure that all children received positive evaluations and avoided any situations that might harm them.

During the test, the researchers uniformly described the gamification features of “Coding Adventure” to children, weakening the concepts of “education” and “tool” to enhance their interest in engagement. Therefore, the “game” expressed by children below refers to this prototype. After the test was completed, the researchers distributed questionnaires to all children who completed the game and collected them. Finally, 312 questionnaires were obtained, of which 295 were complete and met the requirements for data analysis. In the subsequent qualitative interview phase, the researchers randomly selected twelve children who completed the questionnaire as interviewees.

4.3. Data collection and analysis

### 4.3.1. Quantitative survey

In the quantitative survey, the researchers designed survey items based on previous studies and used the cartoon shapes in the prototype as the design background to create a colourful paper questionnaire. The researchers distributed questionnaires to the children to answer after they completed the prototype experience. The children who completed the questionnaire carefully received cartoon stickers as small rewards. The items about “gamification” in the questionnaire were developed from the study of Grangeia et al. (2019), the items about “flow” were developed from the study of Thomas and Baral (2023), and the items about “learning engagement” were developed from the study of Yang et al. (2021). In order to conform to children’s understanding and response habits, the researchers have simplified and adjusted the expression of items. English items were translated into Chinese during the survey and verified by professional translators.

Since the children engaging in this test are concentrated in the second to fourth grades of primary school, the age range of the survey participants is 8-10 years old. The gender ratio of the participants is balanced, and most of them have no experience in coding education. See the Table 2 below for specific data.

**Table 2.** Survey Demographic Profile

| **Category** | **Option** | **Total N=295** | |
| --- | --- | --- | --- |
|  |  | **Frequency** | **Percentage** |
| Age | 8 | 63 | 21.36% |
|  | 9 | 91 | 30.85% |
|  | 10 | 141 | 47.80% |
| Gender | Male | 151 | 51.19% |
|  | Female | 144 | 48.81% |
| Coding experience | Never | 198 | 67.12% |
|  | Less than one year | 76 | 25.76% |
|  | One year and above | 21 | 7.12% |

### 4.3.2. Qualitative interview

After completing the quantitative survey, the researchers designed interview questions based on the results of the analysis. In order to obtain more feedback from children, the interview questions focused on children’s views on gamification design elements (role-playing, reward, challenge and cooperation) during the prototype testing process, avoiding the “flow” and “learning engagement” themes, which are difficult for children to understand.

The interview participants were selected from the children who completed the questionnaire—a total of twelve children. The researchers conducted face-to-face interviews with each of them for about 15 minutes. Participants were coded according to standards for systematic interview analysis and to protect their privacy (Rädiker & Kuckartz, 2020). See the Table 3 below for specific information.

**Table 3.** Interview Demographic Profile

| **Code** | **Gender** | **Age** | **Coding Experience** | |
| --- | --- | --- | --- | --- |
| IK01 | Female | 10 | Less than one year |  |
| IK02 | Male | 8 | Never |  |
| IK03 | Male | 9 | Never |  |
| IK04 | Female | 9 | One year and above |  |
| IK05 | Male | 10 | Never |  |
| IK06 | Male | 10 | Less than one year |  |
| IK07 | Female | 8 | Never |  |
| IK08 | Male | 10 | Never |  |
| IK09 | Female | 9 | Less than one year |  |
| IK10 | Male | 10 | Never |  |
| IK11 | Female | 10 | Never |  |
| IK12 | Female | 10 | One year and above |  |

5. Results

Following the research process of the explanatory sequential mixed method, this study successively displays the results of quantitative surveys and qualitative interviews, and finally integrates and displays them to achieve the effect of explanation. Quantitative data was analysed with partial least squares structural equation modelling (PLS-SEM) using Smart-PLS software, while qualitative data was analysed with thematic analysis (TA) using MAXQDA software.

5.1. Survey results

### 5.1.1. Measurement model

Table 4 focuses on factor loadings, Cronbach’s alpha (CA), composite reliability (CR) and average variance extracted (AVE) in the measurement model. The fact loading of all items is greater than 0.708, the CA of all variables remains between 0.7-0.95, and the AVE exceeds 0.5, which shows the validity of items and variables (Hair et al., 2019).

**Table 4.** The Measurement Model Assessment Results

| **Construct** | **Item** | **Loading** | **CA** | **CR (rho_a)** | **CR (rho_c)** | **AVE** |
| --- | --- | --- | --- | --- | --- | --- |
| Role-playing (RP) | RP1 | 0.918 | 0.940 | 0.940 | 0.957 | 0.848 |
|  | RP2 | 0.936 |  |  |  |  |
|  | RP3 | 0.917 |  |  |  |  |
|  | RP4 | 0.912 |  |  |  |  |
| Reward (RE) | RE1 | 0.830 | 0.831 | 0.836 | 0.887 | 0.663 |
|  | RE2 | 0.817 |  |  |  |  |
|  | RE3 | 0.790 |  |  |  |  |
|  | RE4 | 0.819 |  |  |  |  |
| Challenge (CH) | CH1 | 0.848 | 0.918 | 0.919 | 0.942 | 0.803 |
|  | CH2 | 0.932 |  |  |  |  |
|  | CH3 | 0.923 |  |  |  |  |
|  | CH4 | 0.880 |  |  |  |  |
| Cooperation (CO) | CO1 | 0.892 | 0.926 | 0.930 | 0.947 | 0.818 |
|  | CO2 | 0.935 |  |  |  |  |
|  | CO3 | 0.928 |  |  |  |  |
|  | CO4 | 0.860 |  |  |  |  |
| Flow (FL) | FL1 | 0.892 | 0.898 | 0.913 | 0.925 | 0.714 |
|  | FL2 | 0.905 |  |  |  |  |
|  | FL3 | 0.897 |  |  |  |  |
|  | FL4 | 0.712 |  |  |  |  |
|  | FL5 | 0.804 |  |  |  |  |
| Learning engagement (LE) | LE1 | 0.944 | 0.940 | 0.952 | 0.957 | 0.848 |
|  | LE2 | 0.950 |  |  |  |  |
|  | LE3 | 0.944 |  |  |  |  |
|  | LE4 | 0.841 |  |  |  |  |
| Note: CA = Cronbach’s alpha; CR = Composite reliability; AVE = Average variance extracted | | | | | | |

The researchers applied two criteria to detect the discriminant validity of variables, namely the Fornell-Larcker criterion and the heterotrait-monotrait (HTMT) criterion. Table 5 shows the discriminant validity of the latent variables in this study using the Fornell-Larcker criterion, which requires that the square root of the AVE of the latent variable is greater than the correlation coefficient between the latent variable and all other latent variables (Hair Jr et al., 2021; Sarstedt et al., 2021). Table 6 also verifies the discriminant validity of the variables, with all values being less than 0.85 (Hair Jr et al., 2021; Sarstedt et al., 2021). Through the data verification of Tables 5 and 6, there is no overlap or unclear distinction between the variables in this study.

**Table 5.** Discriminant Validity (Fornell-Larcker Criterion)

|  | **RP** | **RE** | **CH** | **CO** | **FL** | **LE** |
| --- | --- | --- | --- | --- | --- | --- |
| **RP** | 0.921 |  |  |  |  |  |
| **RE** | 0.658 | 0.814 |  |  |  |  |
| **CH** | 0.457 | 0.273 | 0.896 |  |  |  |
| **CO** | 0.464 | 0.256 | 0.733 | 0.904 |  |  |
| **FL** | 0.493 | 0.372 | 0.404 | 0.464 | 0.845 |  |
| **LE** | 0.511 | 0.406 | 0.429 | 0.563 | 0.646 | 0.921 |

**Table 6.** Discriminant Validity (HTMT Criterion)

|  | **RP** | **RE** | **CH** | **CO** | **FL** | **LE** |
| --- | --- | --- | --- | --- | --- | --- |
| **RP** |  |  |  |  |  |  |
| **RE** | 0.743 |  |  |  |  |  |
| **CH** | 0.490 | 0.309 |  |  |  |  |
| **CO** | 0.498 | 0.290 | 0.795 |  |  |  |
| **FL** | 0.531 | 0.413 | 0.445 | 0.505 |  |  |
| **LE** | 0.537 | 0.452 | 0.457 | 0.600 | 0.695 |  |

### 5.1.2. Structural Model

In the structural model of this study, H1, H4, H5, H7, and H9 are proven to have significant positive effects because their *p*-values are less than 0.05 and their *β* values are positive (Hair et al., 2019). Moreover, the *t*-values of these established hypotheses are all greater than 1.96, which indicates that they are significant at the 95% confidence level (Yahaya, 2019). Therefore, the quantitative results show that role-playing and cooperation in gamification elements have significant positive effects on children’s flow experience, and flow experience has a significant positive effect on children’s learning engagement. In addition, rewards and cooperation have been proven to have significant positive effects on children’s learning engagement. (Table 7)

**Table 7.** Hypotheses Testing Results

| **Hypothesis** | | **Std. beta (*β*)** | **Std. dev** | ***t*-value** | ***p*-value** | **Discussion** |
| --- | --- | --- | --- | --- | --- | --- |
| H1 | Role-playing -> Flow | 0.265 | 0.080 | 3.393 | 0.001 | Accept |
| H2 | Reward -> Flow | 0.114 | 0.066 | 1.677 | 0.094 | Reject |
| H3 | Challenge -> Flow | 0.056 | 0.075 | 0.659 | 0.510 | Reject |
| H4 | Cooperation -> Flow | 0.274 | 0.082 | 3.327 | 0.001 | Accept |
| H5 | Flow -> Learning engagement | 0.426 | 0.068 | 6.199 | 0.000 | Accept |
| H6 | Role-playing -> Learning engagement | 0.100 | 0.077 | 1.311 | 0.190 | Reject |
| H7 | Reward -> Learning engagement | 0.114 | 0.057 | 1.997 | 0.046 | Accept |
| H8 | Challenge -> Learning engagement | -0.072 | 0.083 | 0.824 | 0.410 | Reject |
| H9 | Cooperation -> Learning engagement | 0.343 | 0.092 | 3.712 | 0.000 | Accept |

The researchers also examined the value of *f ²* to determine the effect size of established hypotheses (Hair et al., 2019). This study applied the measurement standards for effect size (*f ²*) proposed by Kenny (2016): large (0.025), medium (0.01) and small (0.005). According to the above standards, excluding the influence of reward on children’s flow experience, which can be judged as medium, the influence of role-playing and cooperation on flow, the influence of flow on learning engagement, and cooperation on learning engagement can all be judged as large. See the Table 8 below for details.

**Table 8.** The Effect Sizes of Hypotheses

| **Hypothesis** | | **Std. beta (*β*)** | ***p*-value** | ***f ²*** | **Effect Size** |
| --- | --- | --- | --- | --- | --- |
| H1 | Role-playing -> Flow | 0.265 | 0.001 | 0.050 | Large |
| H4 | Cooperation -> Flow | 0.274 | 0.001 | 0.049 | Large |
| H5 | Flow -> Learning engagement | 0.426 | 0.000 | 0.260 | Large |
| H7 | Reward -> Learning engagement | 0.114 | 0.046 | 0.015 | Medium |
| H9 | Cooperation -> Learning engagement | 0.343 | 0.000 | 0.104 | Large |

The researchers detected the indirect effects in the structural model to determine the mediating effects of the “flow” variable. Table 9 shows that the “flow” variable has significant positive mediating effects (*β* > 0, *p*-value < 0.05) in the relationship between “role-playing”, “cooperation” and “learning engagement” (Hair et al., 2019).

**Table 9.** Mediating Effects Testing Results

| **Mediating Effect** | **Std. beta (*β*)** | **Std. dev** | ***t*-value** | ***p*-value** | **Discussion** |
| --- | --- | --- | --- | --- | --- |
| Role-playing -> Flow -> Learning engagement | 0.113 | 0.039 | 2.967 | 0.003 | Accept |
| Reward -> Flow -> Learning engagement | 0.049 | 0.030 | 1.559 | 0.119 | Reject |
| Cooperation -> Flow -> Learning engagement | 0.117 | 0.040 | 2.870 | 0.004 | Accept |
| Challenge -> Flow -> Learning engagement | 0.024 | 0.033 | 0.637 | 0.524 | Reject |

5.2. Interview results

In this qualitative study, the researchers classified and linked the data collected during the interviews according to the themes of this study, and concentrated the representative quotes(Rädiker & Kuckartz, 2020). Considering the children’s cognitive level, the themes in this qualitative analysis only focus on the gamification elements and do not involve their flow experience or learning engagement. However, the researchers still learned about children’s feedback on flow experience and learning engagement through additional questions, which is reflected in the following results integration part.

5.3. Results Integration

This study conducts joint display through the pillar integration process (PIP) (Johnson et al., 2019). The results of this study are placed in the middle of the table as “pillar themes”, with quantitative and qualitative data presented on either side of it for comparison and presentation (Johnson et al., 2019) (Table 10).

**Table 10.** Pillar Integration of Quantitative and Qualitative Data

| **QUAN** | **QUAN** | **Pillar**  **Building**  **Themes** | **QUAL** | **QUAL** |
| --- | --- | --- | --- | --- |
| **Data** | **Category** |  | **Category** | **Data** |
|  |  |  |  |  |
| *β* = 0.265,  *t* = 3.393,  *p* = 0.001  *f ²* = 0.050 | Significant positive effect at the 95% confidence level  Effect size is large | Role-playing positively influences children’s flow experience in the coding education tool. | Heroic fantasy | “Playing as a knight in the game makes me feel like a hero saving the world.” (IK07) |
|  |  |  | Real fight | “It was impossible for me to actually hold a weapon in the games I played before, but your game makes me feel very real.” (IK05) |
|  |  |  | Easily remember | “After playing it in a real environment, I can easily remember the process of passing the level.” (IK01) |
|  |  |  | Immersion | “Playing such a large game on the playground made me forget about the outside world, as if I was really in the game.” (IK03) |
| *β* = 0.274,  *t* = 3.327,  *p* = 0.001  *f²* = 0.049 | Significant positive effect at the 95% confidence level  Effect size is large | Cooperation positively influences children’s flow experience in the coding education tool. | Teamwork | “If I have any questions, I feel reassured that I can discuss them with my classmates, so I can think about how to pass the test with peace of mind.” (IK02) |
|  |  |  | Communica-tion | “In the past, coding games were played by one person on the computer, and it was boring without being able to chat with classmates. I hope my friends can see what I do.” (IK09) |
|  |  |  | Encourage-ment | “My classmates cheered me on so that I could happily concentrate on the game.” (IK03) |
| *β* = 0.426,  *t* = 6.199,  *p* = 0.000  *f²* = 0.260 | Significant positive effect at the 95% confidence level  Effect size is large | The flow experience positively influences children’s learning engagement in the coding education tool. | Immersion | “Being immersed in the game makes me feel that time passes very quickly, and I feel that I have learned a lot of coding skills.” (IK04) |
|  |  |  | Feeling upgrade | “Through character upgrading in the game, I can feel the role of coding. I must plan my growth route in order to better eliminate enemies.” (IK06) |
|  |  |  | Practicality | “I can feel that the skills in the game are useful. After I learn to use these skills, I can actually use these skills in coding software.” (IK04) |
|  |  |  | Game goal | “The setting of game goals allowed me to understand the process of learning coding from simple to difficult. I can now understand how complex games are made.” (IK07) |
|  |  |  | Teamwork | “It feels good to play games with my classmates. I also hope that I can often cooperate with my classmates to complete something while studying.” (IK10) |
|  |  |  | Good suggestion | “During the game, I learned that coding can also be a matter of brainstorming, and my classmates gave me a lot of good ideas. I think I can use this method in my study.” (IK01) |
| *β* = 0.114,  *t* = 1.997,  *p* = 0.046  *f²* = 0.015 | Significant positive effect at the 95% confidence level  Effect size is Medium | Rewards positively influence children’s learning engagement in the coding education tool. | Level award | “I really like games where you can get rewards for learning. I will keep upgrading and learning.” (IK03) |
|  |  |  | Practicality | “The skills I got after every level in the game are very useful and I think I can use them in Scratch on my pad.” (IK12) |
|  |  |  | Physical prize | “If I get good grades, my family will give me gifts. In order to get the gifts, I will study hard.” (IK02) |
| *β* = 0.343,  *t* = 3.712,  *p* = 0.000  *f ²* = 0.104 | Significant positive effect at the 95% confidence level  Effect size is large | Cooperation positively influences children’s learning engagement in the coding education tool. | Teamwork | “Through cooperation with classmates, I realize the power of teamwork, and I will apply this to my studies.” (IK04) |
|  |  |  | Good suggestion | “But when I encounter difficulties in the game, my classmates will give me suggestions, which I find very useful. I will also ask my classmates for their suggestions when I study in the future.” (IK06) |
|  |  |  | Communica-tion | “It’s so fun to play games and chat in class. I think this is good, as it allows me to quickly learn coding knowledge.” (IK09) |
|  |  |  | Encourage-ment | “When I encountered difficulties, my classmates cheered me on and encouraged me so that I could try new solutions again.” (IK07) |
| *β* = 0.113,  *t* = 2.967,  *p* = 0.003 | Significant positive effect at the 95% confidence level | Children’s flow experience mediates the relationship between role-playing and learning engagement. | Heroic fantasy | “I like playing the role of a hero in games, which allows me to seriously engage in the game environment and learn useful knowledge.” (IK05) |
|  |  |  | Easily remember | “Role-playing allows me to clearly remember all the game processes, so I also remember all the coding skills I learned.” (IK10) |
|  |  |  | Immersion | “I love playing this game, it makes me forget I’m in school, but I remember all the skills.” (IK11) |
| *β* = 0.117,  *t* = 2.870,  *p* = 0.004 | Significant positive effect at the 95% confidence level | Children’s flow experience mediates the relationship between cooperation and learning engagement. | Teamwork | “Cooperating with my classmates allows me to better experience the levels of the game. This teaches me that when I encounter problems in my studies, I can also think of working with my classmates to solve them.” (IK12) |
|  |  |  | Good suggestion | “When I was playing games, my classmates told me a lot of good ideas, which helped me forget the difficulties and pass the levels better. If I encounter problems in my studies in the future, I will ask my classmates more.” (IK06) |
|  |  |  | Communica-tion | “This game gave me and my classmates a common topic. It turns out that so many people like coding. We had a lot of fun playing together. I hope we can play like this in other courses.” (IK08) |

The interviews for the qualitative part were arranged in a short time after the children participated in the game, so as to restore the children’s intuitive feelings as much as possible. This arrangement also brought problems, which is that the excitement brought by the game made the children’s feedback mostly positive. These qualitative feedbacks facilitated the researchers to explain the positive quantitative results, but also brought difficulties to the negative quantitative interpretation. Nevertheless, these feedbacks still contain a certain number of possible reasons for the falsified hypotheses, especially the reason why the role of the “challenge” gamification element was denied in all hypotheses. Therefore, the researchers also specially sorted out the interviewees’ views on the failure of “challenge” in the prototype (Table 11), which will be discussed below.

**Table 11.** Pillar Integration of Invalid Gamification Element in Prototype

| **QUAN** | **QUAN** | **Pillar**  **Building**  **Themes** | **QUAL** | **QUAL** |
| --- | --- | --- | --- | --- |
| **Data** | **Category** |  | **Category** | **Data** |
|  |  |  |  |  |
| *β* = 0.056,  *t* = 0.659,  *p* = 0.510 | Challenge does not influence children’s flow experience in the coding education tool. | Challenge has no effect in this prototype. | Low difficulty setting | “This game is not difficult. I passed it in three attempts. I think you can always pass it if you keep trying. The monsters in the later levels look powerful, but they are actually easy to defeat.” (IK02) |
| *β* = -0.072,  *t* = 0.824,  *p* = 0.410 | Challenge does not influence children’s learning engagement in the coding education tool. |  | Encourage cooperation rather than confrontation | “I feel that this game requires us to work together with our classmates to pass the levels, rather than competing with each other to see who is better, so I don’t have to think about competing with my classmates.” (IK05) |
| *β* = 0.024,  *t* = 0.637,  *p* = 0.524 | Children’s flow experience does not mediate the relationship between challenge and learning engagement. |  | Lack of clear victory goal | “I'm not sure what it means to win. Is it defeating all the monsters, finding the treasure, or getting out of the maze? Or all of them? It would be nice if I could tell it to you clearly at the beginning.” (IK07) |

6. Discussion

Based on the above quantitative and qualitative results, clear discussions can be summarized. Overall, role-playing and cooperation effectively improve the flow experience in coding education tools, and the flow experience effectively promotes children’s learning engagement. Moreover, rewards and cooperation also directly positively influence children’s learning engagement. In addition, flow experience has mediating effects on children’s learning engagement through role-playing and cooperation. In the following, separate discussions are described in more detail based on the specific themes presented above.

The first theme highlights the importance of role-playing in play experiences. Specifically, role-playing shows a positive and efficient effect on children’s flow experience in the learning process. The main reason is that role-playing allows children to experience the feeling of being heroes engaging in “real fights. ” Respondents felt that “playing as a knight” (IK07) “actually holding a weapon in the game” increased the realism of role-playing (IK05). In this process, children more clearly remember the operations performed and information obtained during the engagement process, and are immersed in the gamified environment, even temporarily forgetting the limitations of space and time. The large effect size in quantitative results also further demonstrates the strong influence of role-playing. This is enough to prove that role-playing can indeed stimulate children’s imagination, attract them into the game world, and enable them to obtain an immersive gaming experience (Shroff et al., 2019; Wallius et al., 2023).

The second theme summarizes the importance of cooperation in play experience. Specifically, cooperation positively and efficiently influences children’s flow experience in the learning process. Respondents pointed out that “discussions with classmates during the game” (IK02) provided them with opportunities for communication (IK09) and encouragement (IK03). The main reason is that cooperation provides children with opportunities to engage in teamwork and communicate with their classmates. In addition, a good cooperation mechanism allows children to receive encouragement and suggestions from others once they encounter difficulties in practice. In the quantitative results on effect size, teamwork and communication in multiplayer cooperative games have been proven to be highly effective in stimulating group flow among gamers (Kaya, 2016), thereby stimulating higher levels of emotion and decision-making challenges (Bian et al., 2023).

In the third theme, the importance of flow experience is focused. Specifically, children’s flow experience of the coding education tool positively and efficiently influences their learning engagement. “Being immersed in the game” (IK04) and “feeling character upgrading” (IK06) drove respondents’ application of practical skills (IK04), setting of learning goals (IK07) and building of teamwork (IK01; IK10). The main reasons are the immersion, upgrade experience, practicality, goal-orientedness, teamwork and partner’s suggestions brought by the gamification elements. In the flow experience, children gain a high sense of immersion and presence (Bodzin et al., 2020), which enhances their intrinsic interest and curiosity, thus enhancing their learning motivation (Ji & Shin, 2019). Moreover, the results of the quantitative part prove that the effect of such flow experience is positive and large.

Through the fourth theme, the positive effects of rewards are summarized. Specifically, rewards positively promote children’s learning engagement with coding education tools, but the effect is medium. “Getting rewards through learning” enables the participant (IK03) to “keep upgrading and learning.” Children believe that the virtual rewards brought by upgrades and the practicality and physical rewards brought by skill improvement effectively strengthen their enthusiasm for learning (IK02; IK12). The inner pleasure that children gain from improving their learning activities is regarded as intrinsic rewards, while promotion from the outside world and direct financial rewards are regarded as extrinsic rewards, both of which can be key elements in influencing learning (Huang & Yang, 2021).

The fifth theme also adds the positive role of cooperation in learning engagement. Specifically, cooperation positively promotes children’s learning engagement with coding education tools, and the effect is large. “Playing games and chatting in class” (IK09) brought “teamwork,” (IK04) “helpful suggestion,” (IK06) and “encouragement” (IK07) to children, which allowed them to “quickly learn coding knowledge.” (IK09) Engaging in teamwork and receiving encouragement and suggestions from partners are seen by children as improving their motivation to learn. Engaging in learning activities together can increase the social intimacy between learners, thereby influencing neural synchronisation between each other and generating learning consensus (Pan et al., 2023).

The sixth theme illustrates the mediating effect of children’s flow experience on the relationship between role-playing and learning engagement. Considering the children’s expressive ability, the researchers did not include questions about mediating effects in the interviews, but they still found some clues from the children’s responses as explanations for this part of the discussion. “Playing the role of a hero in game” allows the respondent (IK05) to “seriously engage in the game environment and learn useful knowledge”. “Role-playing allows” the respondent (IK10) to “clearly remember all the game processes” and “the coding skills.” Therefore, the immersion brought by playing heroes allows children to effectively memorise knowledge points in teaching, which makes flow experience a mediator for role-playing to promote learning engagement.

The last theme states the mediating effect of children’s flow experience on the relationship between cooperation and learning engagement. “Cooperating with classmates in the game” taught the participant (IK12) collaborative problem-solving skills in coding learning. Overall, the communication and suggestions brought about by teamwork can make flow experience better play a mediating effect in the process of cooperation promoting learning engagement (IK06; IK08). The discussions of mediating effects are also supported by the research conclusions of Thomas and Baral (2023).

The researchers also explored the reasons why “challenge” as a gamification element failed in this prototype through an additional theme. When designing the initial prototype, “challenge” was considered to be a gamification element with motivational functions. However, the quantitative analysis results showed that “challenge” did not show significant effects in all hypotheses, and the respondents ultimately believed that the difficulty setting of this prototype was too low (IK02), confrontation was not encouraged (IK05), and there was a lack of clear victory goals (IK07), which made the “challenge” element fail to play its due role in the participants’ game process. Future developers can re-evaluate the design of “challenge” related functions, correct the difficulty and settings of “challenge”, and improve the user experience of unplugged children’s coding education.

In addition, the results of the qualitative part also highlight the intrinsic relationship between the gamification elements. Role-playing as a hero can often bring a sense of immersion to participants; teamwork can bring communication, suggestions and encouragement; and tangible rewards can bring more practical motivation. These gamification elements usually do not work independently, but are often mentioned in combination by participants. It can be seen that the combined use of these gamification sub-elements can bring diverse development solutions to the unplugged children's teaching system.

7. Conclusion
   7.1. Theoretical significance

Exploring the mediating effect of flow experience on the relationship between gamification and education engagement under the framework of the S-O-R theory has been paid attention to by researchers (Thomas & Baral, 2023). This study explored the applicability of this framework to children’s learning engagement in the context of a gamified coding education tool, and used the explanatory sequential mixed method to further explain the reasons behind the phenomenon. The effects of role-playing and cooperation as stimulus (S) processes under the framework of the S-O-R theory are obvious. In addition, the mediating effect of flow experience under the framework of the S-O-R theory was once again verified by this study, which means that children’s flow experience has a significant effect as an organism (O) process and is generally recognized by children. This proves that coding education tools do need good gamification design to satisfy children’s immersion in order to achieve better teaching results.

7.2. Practical significance

The prototype “Coding Adventure” developed for this study has been tested and works well. While collecting data, it also received positive evaluations from children, which proves that the gamified coding education tool based on the researchers’ design concept has practical significance. The prototype completed hundreds of rounds of level runs in an unplugged environment, and successfully completed field testing with engaging children. During the testing process, the researchers screened out gamification elements that are effective for children, among which role-playing cooperation and rewards were identified as being able to stimulate children’s good gaming experience and thus achieve the purpose of improving learning outcomes. In unplugged coding teaching scenarios, future developers can consider enhancing participants’ sense of immersion and accomplishment by adding safe wearable equipment, increasing physical rewards, and allowing on-site communication, and incorporate knowledge points into the game process. In addition, the researchers also explored the reasons why the “challenge” element in the prototype design did not achieve the original design intention, and gave development suggestions for increasing difficulty, encouraging confrontation, and setting clear victory goals.

7.3. Limitations and future directions

The prototype for this study was developed based on the Western chivalric cultural background, which may lead to cultural cognitive bias in children from primary schools in China, thus affecting the research results. Due to the researchers’ resource limitations, the sampling of this study was concentrated in two primary schools in one city, which resulted in the conclusions of this study not being able to be extrapolated to a regional or national scale. In addition, due to the young age of the students participants, their language expression ability is limited, and they are unable to have an in-depth understanding of virtual concepts such as “flow experience” and “mediating effect”, which makes the qualitative results of this study relatively simple. Overall, the lack of breadth and diversity in this sampling may lead to a lack of representativeness in the research results. In future studies, researchers can continue to improve the functions of the prototype in order to obtain richer test results. Researchers may also consider surveying and interviewing teachers and parents about their views on unplugged coding educational tools for children. In addition, researchers can consider combining gamification with educational tools in other fields (such as art, language, and natural knowledge) to test the practical value of gamified education tools in these fields.

Author contributions

Conceptualisation, H.M. and W.L.; methodology, H.M.; formal analysis, H.M.; visualization W.L.; writing-original draft preparation, H.M. and W.L.; writing-review and editing, W.L.; supervision, H.M. All authors have read and agreed to the published version of the manuscript.

Declaration of interests

The authors declare that they have no known competing financial interests or personal relationships that could have appeared to influence the work reported in this paper.

Data availability statement

The data that supports the findings of this study is openly available in https://figshare.com/ at https://doi.org/10.6084/m9.figshare.24942342.v1

References

Akiba, D. (2022). Computational Thinking and Coding for Young Children: A Hybrid Approach to Link Unplugged and Plugged Activities. *Education Sciences*, *12*(11), 793. https://doi.org/10.3390/educsci12110793

An, Y. (2020). Designing Effective Gamified Learning Experiences. *International Journal of Technology in Education*, *3*(2), 62-69. ERIC Number: EJ1264027

Anunpattana, P., Khalid, M. N. A., Iida, H., & Inchamnan, W. (2021). Capturing potential impact of challenge-based gamification on gamified quizzing in the classroom. *Heliyon*, *7*(12), 1-22. https://doi.org/10.1016/j.heliyon.2021.e08637

Aura, I., Järvelä, S., Hassan, L., & Hamari, J. (2023). Role-play experience’s effect on students’ 21st century skills propensity. *The Journal of Educational Research*, *116*(3), 159-170. https://doi.org/10.1080/00220671.2023.2227596

Babić, S., & Čičin-Šain, M. (2023). Teacher’s Motivation for Applying the Unplugged MEMA Method for Early Programming Teaching. In *2023 46th MIPRO ICT and Electronics Convention (MIPRO)* (pp. 926-929). IEEE. https://doi.org/10.23919/MIPRO57284.2023.10159857

Berry, L. A., & Kowal, K. B. (2022). Effect of Role-Play in Online Discussions on Student Engagement and Critical Thinking. *Online Learning*, *26*(3), 4-21. ERIC Number: EJ1374283

Bian, Y., Zhou, C., Zhang, Y., Liu, J., Sheng, J., & Liu, Y. J. (2023). Focus on Cooperation: A Face-to-Face VR Serious Game for Relationship Enhancement. *IEEE Transactions on Affective Computing*, *2023*, 1-16. https://doi.org/10.1109/TAFFC.2023.3306198

Bitrián, P., Buil, I., & Catalán, S. (2020). Flow and business simulation games: A typology of students. *The International Journal of Management Education*, *18*(1), 100365. https://doi.org/10.1016/j.ijme.2020.100365

Bodzin, A., Junior, R. A., Hammond, T., & Anastasio, D. (2020). An immersive virtual reality game designed to promote learning engagement and flow. In *2020 6th International Conference of the Immersive Learning Research Network (iLRN)* (pp. 193-198). IEEE. http://doi.org/10.23919/iLRN47897.2020.9155132

Carmona-Halty, M., Salanova, M., Llorens, S., & Schaufeli, W. B. (2021). Linking positive emotions and academic performance: The mediated role of academic psychological capital and academic engagement. Current psychology, 40, 2938-2947. https://doi.org/10.1007/s12144-019-00227-8

Chen, P. Z., Chang, T. C., & Wu, C. L. (2022). Class of Oz: role-play gamification integrated into classroom management motivates elementary students to learn. *Educational Studies*, *2022*, 1-16. https://doi.org/10.1080/03055698.2022.2081788

Chou, P. N. (2020). Using ScratchJr to foster young children’s computational thinking competence: A case study in a third-grade computer class. *Journal of Educational Computing Research*, *58*(3), 570-595. https://doi.org/10.1177/0735633119872908

Creswell, J. W. (2014). *Research Design: Qualitative, Quantitative and Mixed Methods Approaches (4th ed.)*. Sage publications 2014. ISBN: 978-1-4522-2609-5 (cloth).

Csikszentmihalyi, M. (1990). *Flow: The Psychology of Optimal Experience*, Harper & Row. USA.

Csikzentimihalyi, M. (1975). *Beyond boredom and anxiety: Experiencing flow in work and play*. San Francisco: Jossey-Bass Publishers. USA.

Dong, C., Cao, S., & Li, H. (2020). Young children’s online learning during COVID-19 pandemic: Chinese parents’ beliefs and attitudes. *Children and youth services review*, *118*, 105440. https://doi.org/10.1016/j.childyouth.2020.105440

Fantazir, K., & Bartley, M. (2021). Role-Playing Gamification Technologies with Adult Learners. *Imagining SoTL*, *1*, 3-24. https://doi.org/10.29173/isotl520

Flores, E. G. R., Mena, J., Montoya, M. S. R., & Velarde, R. R. (2020). The use of gamification in xMOOCs about energy: Effects and predictive models for participants’ learning. *Australasian Journal of Educational Technology*, *36*(2), 43-59. https://doi.org/10.14742/ajet.4818

Folgieri, R., Vanutelli, M. E., Galbiati, P. D. V., & Lucchiari, C. (2019). Gamification and coding to engage primary school students in learning mathematics: A case study. In *Proceedings of the 11th International Conference on Computer Supported Education. 1* (pp. 506-513). SciTePress. https://doi.org/10.5220/0007800105060513

Grangeia, T. D. A. G., De Jorge, B., Cecílio-Fernandes, D., Tio, R. A., & de Carvalho-Filho, M. A. (2019). Learn + fun! social media and gamification sum up to foster a community of practice during an emergency medicine rotation. *Health Professions Education*, *5*(4), 321-335. https://doi.org/10.1016/j.hpe.2018.11.001

Hair Jr, J. F., Hult, G. T. M., Ringle, C. M., Sarstedt, M., Danks, N. P., & Ray, S. (2021). *Partial least squares structural equation modeling (PLS-SEM) using R: A workbook* (pp. 78-80)*.* Springer Nature, Gewerbestrasse, Switzerland. https://doi.org/10.1007/978-3-030-80519-7

Hair, J. F., Risher, J. J., Sarstedt, M., & Ringle, C. M. (2019). When to use and how to report the results of PLS-SEM. *European business review*, *31*(1), 2-24. https://doi.org/10.1108/EBR-11-2018-0203

Hernández, M. I. O., Lezama, R. M., & Gómez, S. M. (2021). Work-in-progress: The road to learning, using gamification. In *2021 IEEE Global Engineering Education Conference (EDUCON)* (pp. 1393-1397). IEEE. https://doi.org/10.1109/EDUCON46332.2021.9453892

Huang, C., & Yang, Y. (2021). Research on the relationships among learning motivation, learning engagement, and learning effectiveness. *The Education Review*, *5*(6), 182-190. https://doi.org/10.26855/er.2021.06.004

Huang, X., Zhang, M., Fang, J., Li, T., Fang, Y., & Gan, L. (2020). The Impact of Parental Involvement on Children’s Academic Performance: A Moderated Mediation Model. *Studies of Psychology and Behavior*, *18*(5), 666. https://psybeh.tjnu.edu.cn/EN/Y2020/V18/I5/666

Huskey, R., Craighead, B., Miller, M. B., & Weber, R. (2018). Does intrinsic reward motivate cognitive control? A naturalistic-fMRI study based on the synchronization theory of flow. *Cognitive, Affective, & Behavioral Neuroscience*, *18*, 902-924. https://doi.org/10.3758/s13415-018-0612-6

Jacoby, J. (2002). Stimulus‐organism‐response reconsidered: an evolutionary step in modeling (consumer) behavior. *Journal of consumer psychology*, *12*(1), 51-57. https://doi.org/10.1207/S15327663JCP1201_05

Ji, H. E., & Shin, H. W. (2019). Young Foreign Language Learners’ Engagement and Motivation in Augmented Reality-based Vocabulary Learning. *Multimedia-Assisted Language Learning*, *22*(3), 9-31. http://doi.org/10.15702/mall.2019.22.3.9

Johnson, R. E., Grove, A. L., & Clarke, A. (2019). Pillar integration process: A joint display technique to integrate data in mixed methods research. *Journal of Mixed Methods Research*, *13*(3), 301-320. https://doi.org/10.1177/1558689817743108

Kaya, O. S., & Ercag, E. (2023). The impact of applying challenge-based gamification program on students’ learning outcomes: Academic achievement, motivation and flow. *Education and Information Technologies*, *28*, 10053-10078. https://doi.org/10.1007/s10639-023-11585-z

Kaye, L. K. (2016). Exploring flow experiences in cooperative digital gaming contexts. *Computers in Human Behavior*, *55*, 286-291. https://doi.org/10.1016/j.chb.2015.09.023

Kenny, D. A. (2016). *Moderation*. Retrieved 6 April 2023, from http://davidakenny.net/cm/moderation.htm

Krath, J., Schürmann, L., & Von Korflesch, H. F. (2021). Revealing the theoretical basis of gamification: A systematic review and analysis of theory in research on gamification, serious games and game-based learning. *Computers in Human Behavior*, *125*, 106963. https://doi.org/10.1016/j.chb.2021.106963

Lee, J., & Junoh, J. (2019). Implementing unplugged coding activities in early childhood classrooms. *Early Childhood Education Journal*, *47*, 709-716. https://doi.org/10.1007/s10643-019-00967-z

Lee, J., Joswick, C., Pole, K., & Jocius, R. (2022). Algorithm design for young children. *Contemporary Issues in Early Childhood*, *23*(2), 198-202. https://doi.org/10.1177/14639491211033663

Legaki, N. Z., Xi, N., Hamari, J., Karpouzis, K., & Assimakopoulos, V. (2020). The effect of challenge-based gamification on learning: An experiment in the context of statistics education. *International journal of human-computer studies*, *144*, 102496. https://doi.org/10.1016/j.ijhcs.2020.102496

Lomos, C., Seineke, U., Kesting, F., & Luyten, J. W. (2023). The Design of Incentive Systems in Digital Game-Based Learning: How Primary School Children Interact with It. *Education Sciences*, *13*(7), 668. https://doi.org/10.3390/educsci13070668

Luo, Y., Chen, F., Zhang, X., Zhang, Y., Zhang, Q., Li, Y., Zhou, Q., & Wang, Y. (2021). Profiles of maternal and paternal parenting styles in Chinese families: Relations to preschoolers’ psychological adjustment. *Children and Youth Services Review*, *121*, 105787. https://doi.org/10.1016/j.childyouth.2020.105787

Luo, Z. (2022). Gamification for educational purposes: What are the factors contributing to varied effectiveness? *Education and Information Technologies*, *27*(1), 891-915. https://doi.org/10.1007/s10639-021-10642-9

Mao, Y., Yang, R., Bonaiuto, M., Ma, J., & Harmat, L. (2020). Can flow alleviate anxiety? The roles of academic self-efficacy and self-esteem in building psychological sustainability and resilience. *Sustainability*, *12*(7), 2987. https://doi.org/10.3390/su12072987

McMahon, J., Gallagher, E. A., Walsh, E. H., & O’Connor, C. (2021). Experiences of remote education during COVID-19 and its relationship to the mental health of primary school children. *Irish Educational Studies*, *40*(2), 457-468. https://doi.org/10.1080/03323315.2021.1932555

Mehrabian, A., & Russell, J. A. (1974). *An approach to environmental psychology*. The MIT Press. ISBN: 9780262130905

Ofosu-Ampong, K. (2020). The shift to gamification in education: A review on dominant issues. *Journal of Educational Technology Systems*, *49*(1), 113-137. https://doi.org/10.1177/0047239520917629

Oliveira, W., Tenório, K., Hamari, J., Pastushenko, O., & Isotani, S. (2021). Predicting students’ flow experience through behavior data in gamified educational systems. *Smart Learning Environments*, *8*(1), 30. https://doi.org/10.1186/s40561-021-00175-6

Pan, Y., Cheng, X., & Hu, Y. (2023). Three heads are better than one: cooperative learning brains wire together when a consensus is reached. *Cerebral Cortex*, *33*(4), 1155-1169. https://doi.org/10.1093/cercor/bhac127

Papadakis, S., & Kalogiannakis, M. (2019). Evaluating the effectiveness of a game-based learning approach in modifying students’ behavioural outcomes and competence, in an introductory programming course. A case study in Greece. *International Journal of Teaching and Case Studies*, *10*(3), 235-250. https://doi.org/10.1504/IJTCS.2019.102760

Park, J. Y. (2020). The mediating effect of learning flow on learning engagement, and teaching presence in online programming classes. *Journal of The Korean Association of Information Education*, *24*(6), 597-606. http://doi.org/10.14352/jkaie.2020.24.6.597

Pianta, R. C., Whittaker, J. E., Vitiello, V., Ruzek, E., Ansari, A., Hofkens, T., & DeCoster, J. (2020). Children’s school readiness skills across the pre-K year: Associations with teacher-student interactions, teacher practices, and exposure to academic content. *Journal of Applied Developmental Psychology*, *66*, 101084. https://doi.org/10.1016/j.appdev.2019.101084

Qian, J., Jiang, X., Ma, J., Li, J., Gao, Z., & Qin, X. (2023). Accompany Children’s Learning for You: An Intelligent Companion Learning System. *Computer Graphics Forum*, *42*(6), e14862. https://doi.org/10.1111/cgf.14862

Qin, X., Zhang, Y., Gu, P., & Lin, L. (2020). The impact of cooperative learning strategies on pupils’ learning engagement in the smart classroom environment. In *Blended Learning. Education in a Smart Learning Environment: 13th International Conference, ICBL 2020, Bangkok, Thailand, August 24–27, 2020, Proceedings 13* (pp. 365-377). Springer International Publishing. https://doi.org/10.1007/978-3-030-51968-1_30

Rädiker, S. & Kuckartz, U. (2020). *Focused analysis of qualitative interviews with MAXQDA: Step by step.* MAXQDA Press, Berlin, Germany. https://doi.org/10.36192/978-3-948768072

Riar, M., Morschheuser, B., Zarnekow, R., & Hamari, J. (2022). Gamification of cooperation: A framework, literature review and future research agenda. *International Journal of Information Management*, *67*, 102549. https://doi.org/10.1016/j.ijinfomgt.2022.102549

Ryan, R. M., & Rigby, C. S. (2020). Motivational foundations of game-based learning. *Handbook of game-based learning*, 153-176. The MIT Press. ISBN: 9780262043380

Sanchez, E., van Oostendorp, H., Fijnheer, J. D., & Lavoué, E. (2020). Gamification. In *Encyclopedia of Education and Information Technologies* (pp. 816-827). Cham: Springer International Publishing. https://doi.org/10.1007/978-3-030-10576-1_38

Sarstedt, M., Ringle, C. M., & Hair, J. F. (2021). Partial least squares structural equation modeling. In *Handbook of market research* (pp. 587-632). Cham: Springer International Publishing. https://doi.org/10.1007/978-3-319-57413-4_15

Shroff, R., Ting, F., & Lam, W. H. (2019). A conceptual framework for immersion and flow in digital game-based learning: An example of a game-based classroom response system. *Ubiquitous Learning*, *12*(3), 41. https://doi.org/10.18848/1835-9795/CGP/v12i03/41-60

Sovacool, B. K., Axsen, J., & Sorrell, S. (2018). Promoting novelty, rigor, and style in energy social science: Towards codes of practice for appropriate methods and research design. *Energy Research & Social Science, 45*, 12-42. https://doi.org/10.1016/j.erss.2018.07.007

Thomas, N. J., & Baral, R. (2023). Mechanism of gamification: Role of flow in the behavioral and emotional pathways of engagement in management education. *The International Journal of Management Education*, *21*(1), 100718. https://doi.org/10.1016/j.ijme.2022.100718

Threekunprapa, A., & Yasri, P. (2020). Unplugged Coding Using Flowblocks for Promoting Computational Thinking and Programming among Secondary School Students. *International Journal of Instruction*, *13*(3), 207-222. https://doi.org/10.29333/iji.2020.13314a

Vieira, V. A. (2013). Stimuli–organism-response framework: A meta-analytic review in the store environment. *Journal of Business research*, *66*(9), 1420-1426. https://doi.org/10.1016/j.jbusres.2012.05.009

Vyas, L. (2022). “New normal” at work in a post-COVID world: work-life balance and labor markets. *Policy and Society*, *41*(1), 155-167. https://doi.org/10.1093/polsoc/puab011

Wallius, E., Klock, A. C. T., & Hamari, J. (2023). From forceful compliance to motivated endorsement: effects of gamification on policy compliance during COVID-19. *Behaviour & Information Technology*, *2023*, 1-19. https://doi.org/10.1080/0144929X.2023.2286344

Wang, M. T., Binning, K. R., Del Toro, J., Qin, X., & Zepeda, C. D. (2021). Skill, thrill, and will: The role of metacognition, interest, and self‐control in predicting student engagement in mathematics learning over time. *Child Development*, *92*(4), 1369-1387. https://doi.org/10.1111/cdev.13531

Wipulanusat, W., Panuwatwanich, K., Stewart, R. A., & Sunkpho, J. (2020). Applying mixed methods sequential explanatory design to innovation management. In *The 10th International Conference on Engineering, Project, and Production Management* (pp. 485-495). Springer Singapore. https://doi.org/10.1007/978-981-15-1910-9_40

Yahaya, M., Murtala, A. A., & Onukwube, H. N. (2019). Partial Least Square Structural Equation Modeling (PLS-SEM): A Note for Beginners. *International Journal of Environmental Studies and Safety Research*, *4*(4), 1-30. https://www.researchgate.net/publication/339433516

Yang, G., Zheng, D., Chen, J. H., Zeng, Q. F., Tu, Y. F., & Zheng, X. L. (2023). The effects of a role-playing-based micro-game strategy on students’ computational thinking, learning engagement and learning motivation. *Interactive Learning Environments*, *2023*, 1-21. https://doi.org/10.1080/10494820.2023.2223239

Yang, J., Peng, M. Y. P., Wong, S., & Chong, W. (2021). How E-learning environmental stimuli influence determinates of learning engagement in the context of COVID-19? SOR model perspective. *Frontiers in Psychology*, *12*, 584976. https://doi.org/10.3389/fpsyg.2021.584976

Zainuddin, Z., & Keumala, C. M. (2021). Gamification concept without digital platforms: A strategy for parents on motivating children study at home during Covid-19 pandemic. *PEDAGOGIK: Jurnal Pendidikan*, *8*(1), 156-193. https://doi.org/10.33650/pjp.v8i1.2174

Zhang, F., Jiang, Y., Ming, H., Ren, Y., Wang, L., & Huang, S. (2020). Family socio‐economic status and children’s academic achievement: The different roles of parental academic involvement and subjective social mobility. *British Journal of Educational Psychology*, *90*(3), 561-579. https://doi.org/10.1111/bjep.12374

Zhao, J., & Li, Y. (2020). Experimental Study on Gamification Teaching of Mathematics Classroom Practice Based on Flow Experience. In *2020 IEEE 2nd International Conference on Computer Science and Educational Informatization (CSEI)* (pp. 36-40). IEEE. https://doi.org/10.1109/CSEI50228.2020.9142485
