# Supplementary material for: Start learning coding without computers? A case study on children’s unplugged gamified coding education tool with explanatory sequential mixed method
Source: PLoS One. 2025 Sep 3;20(9):e0330896. doi: 10.1371/journal.pone.0330896 (PMC12407412; doi:10.1371/journal.pone.0330896)
Supplement: Attachment — Submitted filename: Response_to_Reviewers_auresp_2.docx [file pone.0330896.s006.docx]

Reviewer #1:

1. The analysis of weaker aspects, such as the "challenge" component, lacks depth. Additional reflection on cognitive load, design, or motivation might illuminate these inconsistencies.

Revision: The possible reasons for the weak effect of the "challenge" element in the prototype of this study are further analyzed in the penultimate paragraph of the discussion.

2. Without comparative analysis against existing tools or teacher/parent perspectives, the claims of practical significance could be overstated. Including such comparisons would strengthen the argument.

Revision: Since the research design did not include feedback from teachers and parents, it is not possible to include relevant discussion in the analysis of the results, but this has been added as limitations in Section 7.3 at the end of the paper. Suggestions are also made in the future research directions.

3. The absence of a pre-test/post-test design restricts conclusions on actual learning improvements.

Revision: In the last paragraph of Section 7.3, the researchers added a revision mentioning this limitation and considering it as a future research direction.

Reviewer #3:

1. In general, academic English should be significantly improved throughout the manuscript.

Revision: The parts of the paper that were not written in academic English have been polished.

2. From my perspective, it would be interesting to explain in greater depth what this ‘unplugged gamified coding activity’(Coding Adventure) consists of and how it is developed in the classroom session. It would help the readers to better understand what it consists of, and it would be useful to be able to replicate future educational practices.

Revision: We have added a whole paragraph to section 4.2 describing the game rules and how to build the prototype and added test photos for visual demonstration.

3. The title mentions 'coding without a computer', however it is not understood how this is carried out or the implications about it.

Revision: The main purpose of this article is to show that it is possible to learn coding without a computer, and it is the beginning stage of learning, which is already indicated in the title. In addition, the researchers also further emphasized the possibility of teaching in a real environment without computers in the prototype design description in Section 4.2.

4. Gamification elements have been used, and it is intended to involve students in learning. However, it is important to identify what the learning objective is and what educational topics/content and/or competencies are intended to be worked on and acquired by the students with the application of this prototype and what educational results are to be achieved. Otherwise, it will lack an educational component and will be only a playful prototype.

Revisions: The following teaching concept has been more clearly explained in Section 4.2. According to the common planning of coding education, the researchers designed the introductory coding statements as command cards in the prototype. By repeatedly using command cards in the game, learners are familiar with the basic logic of coding and reasonably plan the number of times these statements are used. With more challenging puzzle tasks brought by level upgrades, learners gradually understand the use of complex coding statements and master the thinking skills to solve complex problems. The coding knowledge acquired through the above training has become the basis for learners to continue learning CT.

5. In addition, the applied activity is sometimes referred to as “gamified coding activity” and sometimes as “unplugged gamified coding activity”. It is not very clear what the unplugged activity and the coding activity consist of, nor their implications in the results of this study, since what is analyzed are the implications of gamification elements.

Revisions: Firstly, the researchers clearly stated in the title of the paper that the research focus is "unplugged" and included it in the title. Secondly, in the penultimate paragraph of the first chapter, regarding the teaching effect of unplugged coding education, the original point of view focused on the gaming effect. After the revision, more discussions from the teaching effect perspective were added.

6. Further information on hypothesis 9 is missing in section 3.

Revisions: The information in hypothesis 9 in Section 3 has been strengthened. Also, because the format of the paper is to cite previous studies first and then summarize and propose hypotheses, the reviewer may not have noticed the citation paragraph before the hypothesis in the original version.

7. It is mentioned that the participants provided verbal informal consent. For future work, it would be interesting to include the bioethical code of the study, to inform the adults in charge of the underage students about the bioethics of the study to be able to participate in it and collect written information approval.

Revisions: Thank you for your suggestion. In fact, follow-up research is already underway.

8. Justify text of tables 1,10 and 11.

Revisions: Already adjusted.

9. In table 4, the acronyms of the constructions are already used, therefore in the next tables (7,9,10 and 11). As a suggestion, maybe it could be useful to use them to avoid large text within tables.

Revisions: They have all been adjusted to ensure that abbreviations are used to improve reading efficiency without affecting comprehension.

10. In Table 10, regarding ‘Pillar building themes’ maybe it could be more aesthetic to organize according to the order mentioned in the study theoretical framework: 1)RP, 2)R, 3)CH, 4)C 5)F and 6)LE.

Revisions: Considering that Table 10 is based on the order of hypothesis numbers, and the order of the hypotheses is arranged according to the order of influence of the SOR theory, the current order is retained. However, the authors added hypothesis numbers to clarify the order and avoid misunderstanding by readers.

11. The items from the quantitative questionnaire should be included. Also, the questions asked in the qualitative interview should also be specified.

Revisions: All the items and questions included in the questionnaire and interview have been included in Appendices A and B.

12. The versions of the software used may be specified.

Revisions: The version number of the analysis software has been added at the beginning of Chapter 5.

13. Regarding the analysis of the data, it might be useful to explain the section corresponding to Table 9 “Results of the tests of mediating effects (How was the analysis performed, and the results obtained? and explain the results). On the other hand, I do not recognize the hypotheses analyzed in these results in Table 9. What hypotheses do they correspond to in the theoretical framework created in Figure 1, since H5 relates only flow to learning engagement, not with specific elements of gamification. In table 9 three variables are analyzed while in the theoretical framework there is no hypothesis that relates to three variables. Is it possible to compare three variables with the T-student test?

Revisions: The measurement of the mediating effect is indeed an extended part of the study, and no hypotheses were proposed before. Considering that the measurement results of this part did not affect the main conclusions of this study, it is considered to be deleted in this version of the revisions to avoid more space affecting the main part of the study and to ensure the rigor of the hypotheses and verifications.

14. It would be interesting to include more references in the different parts of the discussion.

Revisions: More literature citations have been added to the discussion to increase the richness of the discussion.

Reviewer #4:

1. Section 3. Hypothesis 9 doesn't include a description of the importance of such a hypothesis

Revisions: The information in hypothesis 9 in Section 3 has been strengthened. Also, because the format of the paper is to cite previous studies first and then summarize and propose hypotheses, the reviewer may not have noticed the citation paragraph before the hypothesis in the original version.

2. Section 4.1. This section is written in the future tense, but this study has already taken place, please rewrite this section using the correct tense.

Revisions: Section 4.1 has been mostly revised to ensure the use of correct tense and more academic language.

3. Section 4.2. We don't know characteristics of the students that participated in the study, demographics, besides gender and age? It is hard then to find practical implications for those that might share similar background or circumstances.

Revisions: Demographic data for quantitative studies are in 4.3.1, and demographic data for qualitative studies are in 4.3.2.

4. Section 4.3.1 There is no example of the type of questions that were used in the study, no appendix or URL where someone could check the questionnaire. So this is missing.

Revisions: The questionnaire for the quantitative study has been included in Appendix A.

5. Section 4.3.2 No sample of the questions used in the interviews. Again, no external URL or appendix to read the questions you applied in the interviews

Revisions: The interview questions for the qualitative study have been included in Appendix B.

6. Section 5.1.2 F2 for effect size is related mostly as Cohen effect size. Is it possible to use this more standard way to refer to effect sizes?

Revisions: After discussion, the researchers decided to keep the original reference. On the one hand, the standard cited by the researchers is more updated, and on the other hand, it is more in line with the situation of this study.

7. Section 5.2 When the reviewer was reading this section there was no "warning" that indicated the data for the inteview was going to be presented in section 5.3... So there is a missing paragraph that should explain how the interview data was going to be treated.

Revisions: Instructions for coding the data for the qualitative study have been added to Section 5.2, which also presents the coding results and a preview of the final integration with the quantitative data.

8. Section 7.3 does not covers that there is missing information about the background of the students participating in the study. Please review this

Revisions: The student participants’ age, gender, grade level, and coding learning experience are described in quantitative and qualitative demographic tables (Tables 2 and 3).
