# Supplementary material for: Start learning coding without computers? A case study on children’s unplugged gamified coding education tool with explanatory sequential mixed method
Source: PLoS One. 2025 Sep 3;20(9):e0330896. doi: 10.1371/journal.pone.0330896 (PMC12407412; doi:10.1371/journal.pone.0330896)
Supplement: Attachment — Submitted filename: Response_to_Reviewers_auresp_3.docx [file pone.0330896.s008.docx]

Dear reviewer:

Since the revisions are all for the details of the sentences and formats in the manuscript, they are all marked in the manuscript. Please enter the manuscript for review.

Thank you.
