# Supplementary material for: Start learning coding without computers? A case study on children’s unplugged gamified coding education tool with explanatory sequential mixed method
Source: PLoS One. 2025 Sep 3;20(9):e0330896. doi: 10.1371/journal.pone.0330896 (PMC12407412; doi:10.1371/journal.pone.0330896)
Supplement: Attachment — Submitted filename: Response_to_Reviewers_auresp_4.docx [file pone.0330896.s009.docx]

1. Recommendations of the paragraph before the hypothesis: to mention the four gamification elements

Reply: Revised

2. Add a paragraph explaining hypothesis 9

Reply: In the previous revision, we tried to add an extended paragraph to hypothesis 9, but further revision opinions suggested deleting it to keep it on par with other hypothesis development paragraphs. In addition, the authors have discussed and felt that hypothesis 6-9 are of the same level and have similar content, so the current paragraph layout is relatively even. Therefore, we sincerely suggest that the editor consider maintaining the status quo.

3. Formal and academic writing

Reply: Revised

4. Coherence throughout the whole document

Reply: Made the following revision----

- Corrected some minor formatting issues in the manuscript.
- Summarized the comprehensive impact of gamification elements on flow experiences before H1-4 and added an overall description.
- Adjusted the paragraph layout of 4.1 research design.
- Added a complete description of the five-point Likert scale.
